# Glucosylation and Glutathione Conjugation of Chlorpyrifos and Fluopyram Metabolites Using Electrochemistry/Mass Spectrometry

**DOI:** 10.3390/molecules24050898

**Published:** 2019-03-04

**Authors:** Tessema Fenta Mekonnen, Ulrich Panne, Matthias Koch

**Affiliations:** 1 Department of Analytical Chemistry and Reference Materials, Bundesanstalt für Materialforschung und-prüfung (BAM), Richard-Willstätter Str. 11, 12489 Berlin, Germany; mekonnet@hu-berlin.de (T.F.M.); ulrich.panne@bam.de (U.P.); 2 School of Analytical Sciences Adlershof (SALSA), Humboldt-Universität zu Berlin, Unter den Linden 6, 10099 Berlin, Germany

**Keywords:** pesticide, bioconjugation, oxidative metabolism, glutathione, glucosylation, EC/MS

## Abstract

Xenobiotics and their reactive metabolites are conjugated with native biomolecules such as glutathione and glucoside during phase II metabolism. Toxic metabolites are usually detoxified during this step. On the other hand, these reactive species have a potential health impact by disrupting many enzymatic functions. Thus, it is crucial to understand phase II conjugation reactions of xenobiotics in order to address their fate and possible toxicity mechanisms. Additionally, conventional methods (in vivo and in vitro) have limitation due to matrix complexity and time-consuming. Hence, developing fast and matrix-free alternative method is highly demandable. In this work, oxidative phase I metabolites and reactive species of chlorpyrifos (insecticide) and fluopyram (fungicide) were electrochemically produced by using a boron-doped diamond electrode coupled online to electrospray mass spectrometry (ESI-MS). Reactive species of the substrates were trapped by biomolecules (glutathione and glucoside) and phase II conjugative metabolites were identified using liquid chromatography (LC)-MS/MS, and/or Triple time of flight (TripleTOF)-MS. Glutathione conjugates and glucosylation of chlorpyrifos, trichloropyridinol, oxon, and monohydroxyl fluopyram were identified successfully. Glutathione and glucoside were conjugated with chlorpyrifos, trichloropyridinol, and oxon by losing a neutral HCl. In the case of fluopyram, its monohydroxyl metabolite was actively conjugated with both glutathione and glucoside. In summary, seven bioconjugates of CPF and its metabolites and two bioconjugates of fluopyram metabolites were identified using electrochemistry (EC)/MS for the first time in this work. The work could be used as an alternative approach to identify glutathione and glucosylation conjugation reactions of other organic compounds too. It is important, especially to predict phase II conjugation within a short time and matrix-free environment.

## 1. Introduction

Understanding the fate and toxicity profiles of a compound is a crucial step in the development of new agrochemicals or drugs. Most agrochemicals are transformed to their by-products because of different multi-stress effects such as metabolism (in a living organism), photolysis (e.g., UV and sunlight), and/or manmade processes (e.g., waste treatment plants) [1,2]. Some may even transform into more toxic molecules than the parent compounds [3,4]. Meanwhile, directly or indirectly, pesticides are entered into living organisms and transformed to more hydrophilic metabolites through enzymatic catalyzed reactions. Regardless of widely regulation by different authorities, their consumption through food is not avoidable yet. Hence, pesticides could involve in metabolism mechanisms of a living organism.

The metabolic processes are generally divided into two groups; phase I and phase II. In phase I, the parent compound converted to more hydrophilic and more mobile metabolites by insertion of functional groups (like -OH) or unmasking of the hydrophilic functional groups such as *N*-, *O*- & *S*-dealkylation, and hydrolysis [5,6]. Phase I metabolism reactions are mediated primarily by cytochrome (CY) enzyme families (e.g., CYP450) in the liver. However, some phase I products are not eliminated fast instead undergo a subsequent phase II reaction with an endogenous substrate such as glutathione (GSH) and glucoside in order to form typically polar, less mobile, and easily excretable conjugates [7]. This phase II metabolism is often catalyzed by transferase enzymes such as glutathione-S-transferase (GST) and glucuronosyltransferase (UGT). Phase II conjugation is a detoxification process; however, some group of chemicals could resulted in toxic metabolites formation [8]. For instance, GSH conjugation of vicinal dihalogenated compounds produce monosubstituted derivatives which may cyclize into a highly electrophilic episulfonium ion [9]. Additionally, Monks et al. [10] have been discussed four mechanisms of GSH reactions that could lead to bioactivation.

On the other hand, the conventional analytical methods in vitro (e.g., incubation with microsomal assay) and in vivo (laboratory animals) are used for a long time as a model experiment to investigate metabolism processes. However, those models have limitations in terms of cost and time of analysis and matrices complexity [11]. Especially, to investigate phase II metabolism processes the conventional approach is more difficult to identify reaction intermediates that lead to conjugation. Meanwhile, non-biological approaches such as electrochemistry coupled to (EC/MS) and microfluidic devices enable fast, cost-effective, and matrix-free detection of phase II conjugative products and short living intermediates [12,13]. Hence, this work primarily targeted to develop EC/MS method for identification of GSH and glucoside conjugation with chlorpyrifos (CPF, an insecticide) and fluopyram (FLP, a fungicide) metabolites. CPF is an anticholinesterase organophosphate insecticide which detected frequently in the European food web system for the last five years. According to the Rapid Alert System for Food and Feed (RASFF) report, 379 warning incidents of CPF were issued in 2018 alone [14]. On the other hand, FLP is a multispectral fungicide that inhibits succinate dehydrogenase enzyme. Unlike CPF, FLP comes to the market in 2013 and its biotransformation processes and residual occurrences are not studied enough. 

Both CPF and FLP phase I transformation products (TPs) were investigated in our group [15,16]. CPF is metabolized to oxon, diethylthiophosphate, diethylphosphate, and trichloropyridinol [17,18,19,20]. Choi et al. [17] and Fujioka et al. [21] were investigated CPF and its oxon metabolite conjugation with GSH by liver microsome incubation. On the other hand, FLP undergoes phase I metabolism through *N*-dealkylation, hydroxylation, and dechlorination [15,22]. However, its phase II metabolism is not investigated yet. On the other hand, both CPF and FLP or their TPs could enter to living organisms and conjugated with native biomolecules (e.g., protein, glucoside, and nucleic acid). Thus, in this work, the two pesticides’ conjugative products with GSH and glucoside were investigated by EC/MS. Intermediates and phase I oxidative metabolites were produced by a boron-doped diamond (BDD) working electrode (WE) and trapped online by biomolecules and detected on ESI-MS. Phase II conjugates of CPF and FLP were identified by the outlined experiments. The results show that EC/MS could be used as a supplementary method to identify phase II metabolites by saving time and cost of analysis without biological matrices.

## 2. Results and Discussion

Active phase I metabolites are detoxified by phase II conjugative reactions which include GSH conjugation, glucuronidation, glucosylation, sulfation, acetylation, methylation, and amination. This work is dedicated to the identification of GSH-conjugation and glucosylation of CPF and FLP metabolites. The analytical strategies were achieved as follows. Primarily, conjugates of TPs of model compounds were produced by EC flow-through cell either by trapping online with biomolecules (EC/MS) or by infusing the effluents to an Eppendorf tube containing the biomolecules (offline). Additionally, the substrates were incubated with RLM as a reference method. Secondly, the offline EC products and RLM incubates were separated and analyzed by targeted fragmentation (MS^2^ and MS^3^) on LC-QTRAP MS/MS. After comparison of RLM incubates and EC products (by retention time, MS spectra, isotopic pattern and adducts), both products were further investigated using TripleTOF in order to determine the accurate mass of the conjugates. After trapping oxidative stressed products of the intended analytes with biomolecules, several bioconjugates were identified. Furthermore, the conjugates identified by online EC/MS (non-microsomal methods) were well compared with liver microsome incubates. Mass voltammograms were recorded by EC/MS at the specified working potential and the first insights of the molecular ions of the conjugates were obtained online by a single quadrupole ESI-MS. The *m*/*z* traces were mapped based on their retention time to have insights about possible isotopic patterns or adducts that elute at the same retention time. 

### 2.1. Glutathione Conjugation of CPF TPs: Online EC/MS vs. Liver Microsomes

**Figure 1:**
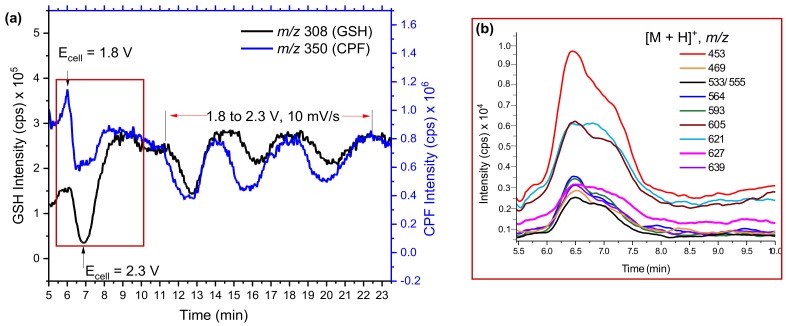
Mass voltammograms of CPF (1800→2300 mV, 10 mV/s) and GSH (0 mV) (**a**) and intensity of possible conjugates (**b**) measured by EC/MS using BDD in µPrepCell^TM^.

Several oxidation products of CPF from P-oxidation and *O*-dealkylation, as well as dechlorination products, were identified by EC/MS or in LMs [16]. The TPs and intermediates (reactive species) were trapped and reacted online in a loop before being entered into the ESI source. As shown in Figure 1a, the mass voltammogram of GSH and CPF were significantly decreased when the potential changed from 1800 mV to 2300 mV in all four subsequent full cycle scans. The reduction in CPF intensity was expected as it was oxidized into TPs at the optimal potential. However, GSH appeared to behave like CPF without any applied potential. This could be explained by the reaction of GSH with some of the TPs of CPF. Online EC/MS enables observation of the reaction products *m*/*z* in real time. Hence, in addition to the TPs of CPF, *m*/*z* 453, 469, 533, 564, 593, 605, 621, 627, 639, and many others were detected in the online EC/MS spectra after being trapped by GSH (Figure 1b). 

**Figure 2:**
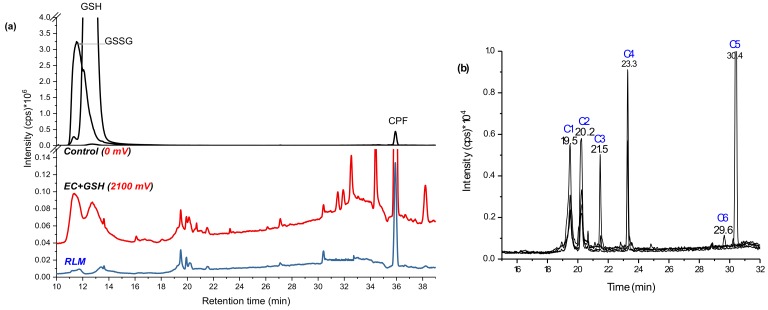
Total ion chromatogram (TIC) of GSH and CPF oxidative products in the control (black), in 2100 mV DC potential (red), and in RLM incubates (blue) (**a**), and extracted ion chromatogram (EIC) of bioconjugates at 2100 mV DC potential (**b**) using BDD WE recorded by LC-MS/MS on (+) ESI.

The extracted mass voltammogram of each *m*/*z* observed between 5.5–10 min (Figure 1b) showed an increase in the time when both CPF and GSH decreased (after the potential applied); this then returned to zero when the potential decreased below 1800 mV. This provides important evidence that the observed *m*/*z* traces may be conjugative phase II products. However, some *m*/*z* traces could be H-bounded adducts or interferences formed during EC reactions, unless they were resolved by chromatographic separation. Therefore, effluents of GSH and TPs mixtures were investigated by offline LC-MS/MS (Figure 2a,b).

The control (black line in Figure 2a), which was the same mixture at zero potential, showed only three distinctive peaks at 11.4 (*m*/*z* 613, [GSSG + H]^+^), 12.6 (*m*/*z* 308, [GSH + H]^+^), and 36.0 min (*m*/*z* 350, [CPF + H]^+^). The peak at 11.4 min was confirmed as GSH-oxidized to GSSG (δm/m = −1.3 ppm in Table 1) by forming a disulfide bond (-S-S-) through the Cys-thiols. It is common for GSH oxidation to GSSG at room temperature [23]. When a potential of 1800–2100 mV was applied (red line in Figure 2a), different peaks were observed and the intensities of both GSH and GSSG decreased. The substrate was then incubated in RLM together with GSH and GST. The supernatant was run on LC-MS alongside the EC effluents (blue line in Figure 2a) and revealed four peaks that matched to the EC/MS products by retention time (19.5, 20.2, 21.5, and 30.4 min) and by the *m*/*z*. Traces of the *m*/*z* observed by online EC/MS experiments (Figure 1b) were investigated using selected ion monitoring (SIM) using the offline LC-MS/MS system. At 19.5 min (conjugate 1: C1) *m*/*z* 487 and at 20.2 min *m*/*z* 469 (C2) were eluted. At 21.5 min C3, *m*/*z* 621 and product ions 605 (−NH_2_), 593 (−CO_2_), and 564 were detected. At 30.4 min C5 elutes with the same *m*/*z* 621 were detected, but without the product ions. Furthermore, at 23.3 min *m*/*z* 605 and 564 (C4) and at 29.6 min *m*/*z* 627 (C6) were separated by LC-MS/MS (Figure 2b).

**Figure 3:**
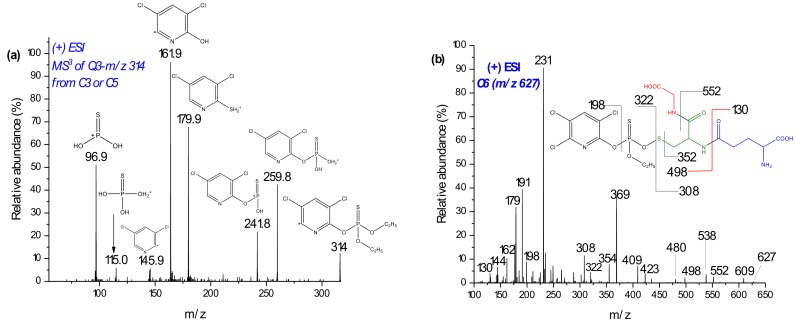
Mass spectra of product ion *m*/*z* 314 from C3 in MS^3^ (**a**) and C6 with its suggested fragmentation measured in MS^2^ (**b**) using LC-QTRAP MS/MS.

When higher deviation (around δm/m = ±5 ppm) of masses on the biomolecules or substrates were observed, the TripleTOF was recalibrated using the calibration solution. In addition to the comparison of retention times, further confirmation was performed for each conjugate using the following two strategies: (1) selective fragmentation of the molecular ion peaks of the conjugate in QTRAP-MS/MS and checking whether the moiety from the expected TPs of the parent compound (plus 34 Da mass (+SH_2_^+^)) was present and; (2) fragmenting the moiety of the substrate, or the biomolecule, in MS^3^ and comparing the fragmentation pattern with the standard. For example, the molecular ion peak at *m*/*z* 621 was expected to be a conjugate of GSH and CPF through loss of neutral HCl. Hence, after fragmentation using QTRAP-MS/MS, product ion *m*/*z* 314 (Q3) was further fragmented at the third quadrupole (Figure 3a). The peaks at *m*/*z* 314, 162, and 146 were lower than the corresponding dechlorinated CPF product by 2 Da. The other peaks also coincided with monodechlorinated CPF [16]. Similarly, the ion product of *m*/*z* 605, and *m*/*z* 298 (oxon–Cl) was analyzed. Thus, C5 could be a GSH and CPF conjugate formed after the loss of a neutral HCl. 

Two products of *m*/*z* 627 (C6 at 29.6 min) and 639 also increased in intensity on EC/MS (Figure 3b). The *m*/*z* 639 chromatogram peak was not found by offline LC-MS/MS investigation. QTRAP-MS/MS experiments and comparison of isotopic distribution with theoretical formula coincided with *m*/*z* 627, as a conjugate of desethyl CPF with GSH. In Figure 3b, the product ions of *m*/*z* 627 at *m*/*z* 322 and 308 may represent the fragmentation of the -O-S- bond. Furthermore, product ions of *m*/*z* 130–231 confirm the presence of GSH. The product ion at *m*/*z* 198 exclusively shows the presence of the pyridine ring. On the other hand, *m*/*z* 354 could be dealkylated CPF plus 34 Da (+SH_2_). Hence, C6 could be a conjugate with GSH and CPF after the loss of C_2_H_6_ (Figure 3b). On the other hand, *m*/*z* 639 yielded product ions at *m*/*z* 308 ([GSH + H]^+^) and 334 ([oxon + H]^+^), along with further characteristic peaks of GSH (*m*/*z* 130–290, a result not shown). However, the conjugates with *m*/*z* 627 and 639 were not postulated since their accurate mass measurement deviations (by TripleTOF-MS) were relatively high and unable to found them in RLM incubates.

**Figure 4:**
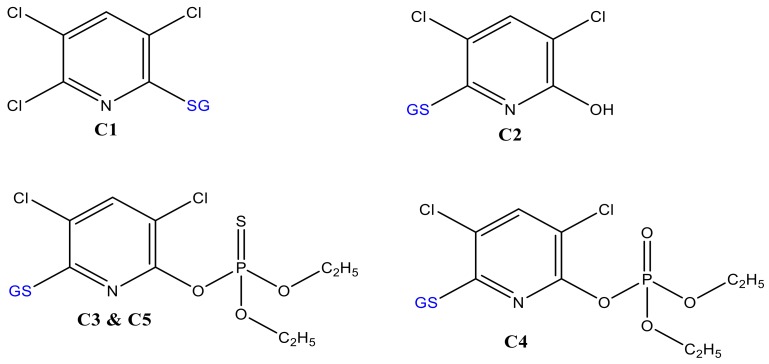
Proposed molecular structures of GSH-conjugates with CPF oxidative products.

Despite the benefit of matrix-free detection, a major issue of simulating conjugative phase II metabolites in such a non-biological system is the difficulty in determining the binding sites between TPs and the trapping agent. Often, the more nucleophile thiol is susceptible to bonding with the substrates. In the case of biological systems, the conjugation sites are more specific and selective because of the respective enzymes. Indeed, C3 and C5 (Figure 4) may be conjugates of CPF at different sites of GSH (via -SH, -NH_2,_ or -NH-) or the three Cl substituted by GS. In the case of trichloropyridinol (TCP), two GSH conjugates were identified at *m*/*z* 487 (C1) and 469 (C2 in Figure 2b). After fragmentation by QTRAP-MS/MS, the absence of product ions at *m*/*z* 155, 171, and 137 confirms that both *m*/*z* 469 and 487 (Table 1) are not TPs containing diethylphosphate (DEP) or diethylthiophosphate (DETP). The conjugate C2 was previously reported by Choi et al. [17]. The peak at *m*/*z* 487 was observed to yield *m*/*z* 180 (TCP–OH) instead of *m*/*z* 162 (TCP–Cl). In addition, the product ions at *m*/*z* 308, 291, 179, 205, and 233 (Table 1) confirmed the presence of GSH. Hence, C1 and C2 (look structures in Figure 4) could be conjugation products of GSH with TCP after loss of water and HCl molecules, respectively.

Many mass traces, including *m*/*z* 407, 453, 533, 564, and 593 were also produced during online EC/MS (their intensities increase with applied potential); however, their chromatographic separation was not successful. Some of the peaks were eluted at the same retention time with GSSG (e.g., *m*/*z* 453, 407, and 533) and with C3 and C4 (e.g., *m*/*z* 564 and 593), which are likely fragmentation products of GSSG in ESI. We, therefore, focused on the peaks that coincided with those from liver microsome incubates. As the formation of conjugates in online EC/MS depends on the reaction conditions (pH, temperature, and organic solvents), more conjugation products can be predicted by EC/MS.

### 2.2. Glucosylation of CPF TPs in Online EC/MS

**Figure 5:**
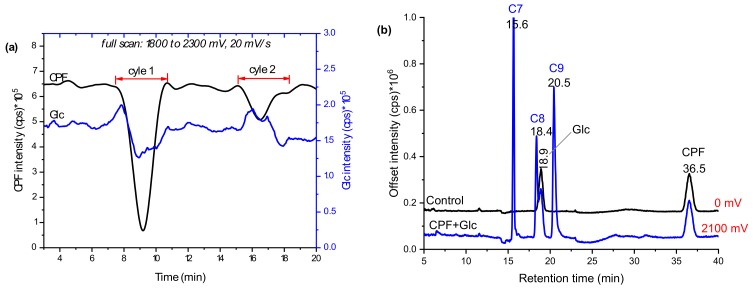
Mass voltammograms of CPF oxidative products and Glc after scanning in 1800–2100 mV using EC/MS equipped with BDD WE (**a**) and EIC chromatograms with and without applied potentials measured by LC-MS/MS (**b**).

**Figure 6:**
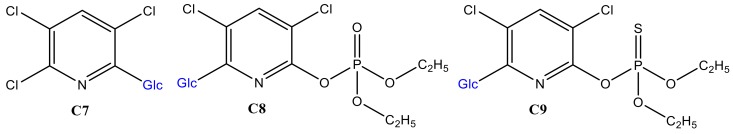
Proposed structures of CPF TPs glucosylation products in online EC/MS.

In addition to GSH conjugation, glucosylation is one of the many phase II metabolism mechanisms for xenobiotics. Pesticides, in particular, often come into direct contact with foodstuffs that contain glucosides. Thus, the glucosylation of CPF TPs was investigated here by trapping with β-d-glucoside (Glc) instead of GSH. As evidenced in Figure 5a, Glc intensity decreased in the first cycle when no potential was applied. In the second cycle, there was low variation in both CPF and Glc intensities, which could either signify adsorption of some products to the BDD surface or that more reaction time was needed before infusing to ESI. Nevertheless, a slight decrease in Glc intensity could also be evidence of conjugate formation. Further separation of the effluents in offline LC-MS/MS revealed an additional three distinctive peaks at 15.6 (C7), 18.4 (C8), and 20.5 min (C9), compared to control (the same composition at zero potential). Similarly, the intensity of Glc at 18.9 min instantly decreased when potential was applied (Figure 5b). The *m*/*z* eluted together with C7 were 360, 362, 364, which confirm Cl-isotopes of the TPs of CPF. Furthermore, C7 did not show fragments with *m*/*z* 153, 137, 97, or 171; however, *m*/*z* 198 (TCP), 180 (TCP-OH), 163 (Glc-OH), and 324 (Table 1) peaks were observed. These findings are suggestive of formation of a conjugate, C7, between TCP and Glc after loss of a water molecule (C7 in Figure 6). On the other hand, C8 with *m*/*z* 478, 480, and 482 were shown to fragment to *m*/*z* 316, 163, 153, 137, and 162 (Table 1). The product ion at *m*/*z* 153 and 137 are characteristic of a dimethylphosphate group, while *m*/*z* 162 with Cl-isotopes suggest the presence of the pyridine ring.

On the other hand, product ions *m*/*z* 316 and 163 may reflect the formation of a dechlorinated oxon with additional ‘O’ and Glc–OH, respectively. Moreover, analysis of the effluents using TripleTOF-MS C8 revealed with δm/m = 6.5 ppm. It is, therefore, reasonable to conclude that C8 is formed between oxon and Glc after the loss of a neutral HCl (C8 in Figure 6). According to Choi et al., the *ortho-*Cl of the pyridine ring is readily cleaved to form a conjugate [17]. Furthermore, C9 appears with a molecular ion peak at *m*/*z* 494 and product ions at *m*/*z* 515 (+Na-adduct), 314 (CPF-Cl), 171 (DETP), and 163 (Glc–OH). Thus, as C8, C9 could be a conjugate of CPF and Glc through the removal of a neutral HCl (look structures in Figure 6). Conformational liver microsome incubation was not performed for Glc due to unavailability of UDP-UGTs enzymes. 

### 2.3. Glutathione Conjugation and Glucosylation of FLP TPs

**Figure 7:**
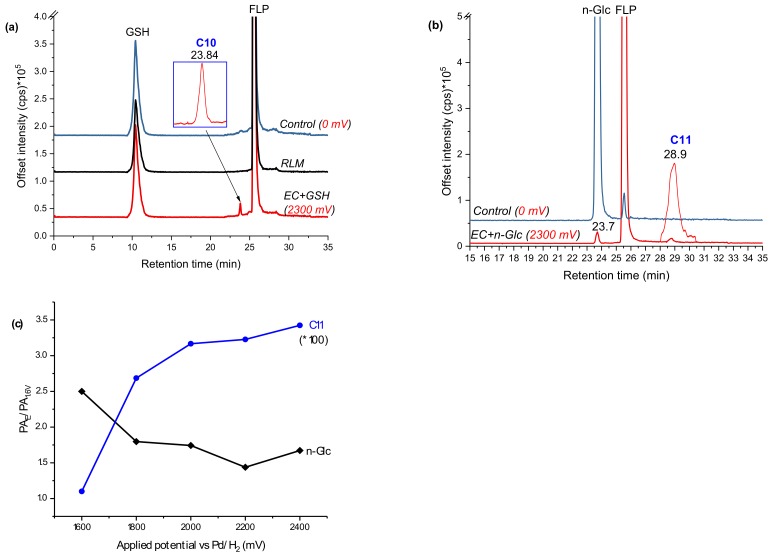
EIC of FLP oxidative products effluent incubated with GSH (**a**) or n-Glc (**b**), and peak area ratios of selected conjugates shown at different applied potentials (**c**), measured by LC-MS/MS on (+) ESI.

**Figure 8:**
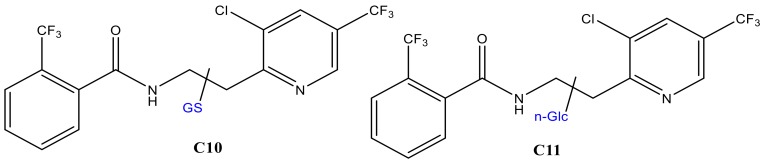
GSH (C10) and glucoside (C11) conjugates of monohydroxyl FLP.

For FLP, similar experiments were performed by trapping the EC effluents with GSH and β-nonyl-glucoside (n-Glc, [M + H]^+^: *m*/*z* 307). Oxidation of FLP was performed using a BDD electrode in the presence of 5% water as a modifier. Only monohydroxyl FLP conjugates with both GSH and Glc was formed. The peak at 23.8 min (C10 in Figure 7a) and 28.9 min (C11 in Figure 7b) appeared at *m*/*z* 702 and 701 after trapping with GSH and n-Glc, respectively. The product ions of C10 (*m*/*z* 702/705) at *m*/*z* 429 (FLP + SH), 395 (FLP–H), 308 (GSH + H), 173 (trifluoromethylbenzoyl ion), 145 (trifluoromethylphenyl), 129, 291, 179, and 162 (Table 1) are characteristic of both GSH and FLP. The conjugate C10 (Figure 8) may have been formed between GSH and monohydroxyl FLP after the loss of H_2_O (nucleophilic substitution) or, between GSH and olefin FLP via addition reaction. However, C10 was not found in the RLM incubations (Figure 7a). Instead, the conjugate, C11 was shown at *m*/*z* 701 with Cl-isotope at *m*/*z* 703. 

The peak area ratio between applied potential ’E’ (PA_E_) and 1600 mV (PA_1.6V_) was significantly increased for C11 and decreased for n-Glc, with increasing potential (Figure 7c). It is experimentally possible to detect a different conjugate by changing the working potential or reaction conditions, as a result of which the TPs would vary accordingly. The product ions were measured at m/z 573 ([M + H]^+^–nonyl), 413 (FLP + OH), 395 (FLP – H^+^), 307 (n-Glc + H^+^), 289 (n-Glc–O), 173 (trifluoromethylbenzoyl ion), 145 (trifluoromethylphenyl), and 129 (n-nonyl) by QTRAP-MS/MS (Table 1). The product ion m/z 395 and 307 could have been produced due to cleavage on the n-Glc–FLP bond, which features proton with n-Glc. On the other hand, the product ion *m*/*z* 413 could represent monohydroxyl FLP, generated upon n-Glc cleavage of the -O-C- bond to yield *m*/*z* 289. Additionally, the peak at *m*/*z* 573 could be explained by n-nonyl (*m*/*z* 129) lost from C11. Thus, C11 could be a conjugate of n-Glc and monohydroxyl FLP following neutral water loss. FLP is also known to form imine and olefin intermediates, which could easily conjugated with n-Glc [24]. Nevertheless, the n-Glc-FLP conjugate formed through N-oxides (on the aliphatic -NH- or pyridine ring -N=) could still produce a molecular ion peak at *m*/*z* 701. With the observation of *m*/*z* 395, the most probable conjugation mechanism of C11 is n-Glc with monohydroxy-FLP through water loss (C11 in Figure 8). Unlike CPF conjugates, several interfering adducts that are not real conjugates were found in the FLP oxidative product with n-Glc. For instance, *m*/*z* 635 (2*n-Glc + Na), 651 (2*n-Glc + K), 725 (n-Glc + FLP + Na), and 815 (2*FLP + Na) were found as interfering adducts (data not shown).

In summary, seven conjugates of CPF oxidative products, four with GSH and three with Glc, and two conjugates of monohydroxyl FLP with GSH and n-Glc were identified using the intended method. The results were compared with RLM incubates. However, the scope of this work did not permit a conclusion on the conjugative sites of the above products (e.g., the sites at which Cl- of CPF, or HO- of Glc were cleaved to form a conjugate bond). Additional conjugates of both compound’s oxidative products with each biomolecule could be identified by modifying the electrochemical reaction (e.g., length and temperature of the reaction loop, chemical composition, and EC cell potential). Analysis time and matrix complexity were highly improved compared to RLM incubation experiments. Investigating bioconjugation of drugs is an important task for many pharmaceutical industries. Furthermore, many agrochemicals could form conjugation with molecules originated from plants such as proteins and glucosides. Hence, this work could be useful in these areas.

## 3. Experimental

### 3.1. Chemicals and Reagents

Analytical standard of CPF (99.7% purity), HPLC-grade acetonitrile (ACN, 99.9%), and methanol (MeOH, 99.85%) were purchased from Th. Geyer (Renningen, Germany). Analytical standard FLP (99.9% purity), reduced GSH (98%), and equine liver GST (74.7% protein) enzyme were obtained from Sigma-Aldrich (Steinheim, Germany). Other chemicals, β-d-glucoside (Glc) and n-nonyl-β-d-glucoside (n-Glc) from Anatrace (Maumee, OH, USA), ammonium formate (NH4FA) from Fluka Chemie (Buchs, Switzerland), and formic acid (HFA) from J.T. Baker (Arnhem, The Netherlands) were purchased in their reagent grades. Ultrapure water was produced by a Seralpur PRO 90 CN system (Ransbach-Baumbach, Germany).

### 3.2. Electrochemical Oxidation and Analysis of TPs

The electrochemical oxidation of the parent compounds was achieved by an electrochemical flow-through cell (µPrepCell^TM^ from Antec Scientific, Zoeterwoude, The Netherlands) based on our previous methods [15,16]. Briefly, a full scan (10 mV/s) potential ranged 1800–2100 mV for CPF (0.1 mmol/L in ACN/MeOH/H_2_O, 20:60:20% *v*/*v*/*v*) and 1650–2500 mV for FLP (0.1 mmol/L in ACN/H_2_O, 95:5% *v*/*v*) were applied. Ammonium formate (1 mmol/L) for CPF and HFA (0.1% *v*/*v*) for FLP were used as an electrolyte to increase the conductivity of the solution. The electrochemical cell was consisting of BDD working electrode (WE), Pd/H_2_ reference electrode (RE), and Ti-block counter electrode (CE). The CE and WE were separated by two 100 µm spacers. Before each measurement, the working electrode was pulsed at E1 = 2, E2 = −2, and E3 = 0 V (for 100 ms on each step) for a total of 5 min and rinsed using solvents without the analyte. Blank samples were performed similarly. The electrochemical products (oxidative products, intermediates, adducts, radicals, and unreactive species) were passed online to ESI source of a single quadrupole-MS (Agilent Technologies GmbH, Waldbronn, Germany). Further characterization, mechanism elucidation, and screening of oxidative products were performed based on Mekonnen et al.’s work [15,16].

### 3.3. Adduct Formation with Biomolecules

The oxidative products of CPF or FLP (from EC cell) were trapped online by a solution of 0.5 mmol/L GSH or glucoside (adjusted to pH 7.2 using NH_4_FA and HFA). The mixtures were allowed to react in a reaction loop (2.25 m) and infused to ESI-MS online. Blank solvents and control samples (a mixture of substrate and trapping agent at zero volt) were run simultaneously. A nominal mass of 100–2000 Da was scanned. The ESI-MS conditions were sated at +5000 ionization source (IS), 13 L/min drying gas flow, 60 psig nebulizer gas pressure, and 350 °C drying temp. For furthermore structural elucidation by LC-QTRAP MS/MS and TripleTOF-MS, oxidative products were infused into an Eppendorf tube containing 0.5 mmol/L biomolecule (pH 7.2). The mixtures were vortexed (for 1 min) and incubated at 800 rpm, 37 °C for 2 h before LC-MS/MS analysis. Additionally, the effect of pH was investigated by adjusting the mixtures (before incubation) at 3.5, 7.4, and 9.2 using NH_4_FA and HFA. To compare with in vitro microsomal assay, the substrates (CPF and FLP) were incubated with RLM by slight modification of Mekonnen et al.’s work [15,16]. All procedures were the same except for the addition of 10 µL GST. Triplicate experiments were performed for both EC/MS and RLM and the reproducibility of the produced conjugates (type of products, not quantity) were checked by visualizing *m*/*z* traces of mass voltammograms. EC effluents and RLM incubates (150 µL) were investigated by LC-MS/MS simultaneously.

### 3.4. Offline LC-MS/MS Analysis of Bioconjugates

The incubated supernatant mixture (150 µL) was analyzed by an Agilent 1200 series LC hyphenated to an AB Sciex 4000 QTRAP^®^ MS/MS (Foster City, CA, USA). Luna Omega Polar C18 (250 × 4.6 mm, 5 µm dimension) from Phenomenex (Aschaffenburg, Germany) was used as an analytical column. The mobile phases were water (A) and MeOH (B) both with 5 mmol/L NH_4_FA in case of CPF and water (A) and ACN (B) both with 0.1% HFA in case of FLP conjugates. After gaining information on the probable conjugative products from online EC/MS, targeted precursor ions were fragmented on enhanced product ion scan (EPI) in (+) ESI-MS/MS by direct infusion. MS/MS spectra were acquired for 3 min with 4000 Da/s scan rates, 7 s delay time, +5000 V IS, 35 psi curtain gas (CUR), 500 °C source temp., 200 ms dwell time, 20 psi source gas 1 (GS1), 60 psi source gas 2 (GS2), +100 eV declustering potential (DP), +45 eV collision energy (CE) with 5 eV spread (CES), and high CAD. Data was acquired using Analyst^®^ 1.5.2 software (AB Sciex). The flow rate of the mobile phase was 250 µL/min with 5 µL injection volume and 45 °C column compartment temperature. The gradients of CPF-derived conjugates were: 80% A linearly switched to 100% B in 20 min, kept for 5 min, linearly switched to 60% A within 5 min, 20% A linearly dropped to 100% B within 10 min, and finally switched to 80% A from 45 to 60 min. The same gradient profile was used in the case of FLP-derived conjugates except 80% A was kept isocratic for the first 15 min.

### 3.5. Confirmation by HRMS

An AB Sciex TripleTOF^®^ 6600 was used to confirm accurate masses of the proposed bioconjugates. The samples were infused at 7 µL/min. For TOF-MS experiments (Q1 scan) the conditions were: +5000 V ion spray voltage floating (ISVF), 400 °C temperature, +100 eV DP, 10 eV CE, 20 psi GS1, 15 psi GS2, 25 psi CUR, and 7 CAD. Ions were accumulated for 2.5 s and scanned in the range of 100–2000. For TOF-MS/MS experiments (product ion scan), the same experimental conditions were used, except for 40 psi GS2, 40 eV CE with 5 eV collision energy spread (CES), 30 ms ion release delay (IRD), and 15 ms ion release width (IRW) were fixed. Data was acquired using Analyst^®^ 1.7.1 (AB Sciex).

## 4. Conclusions

In this work, the application of electrochemistry coupled online to mass spectrometry for investigation of phase II conjugative metabolites of two model pesticides transformation products, chlorpyrifos and fluopyram, was investigated. Chlorpyrifos and its transformation products namely trichloropyridinol and oxon were conjugated with glutathione by losing a neutral HCl. Additionally, three glucosylation products of chlorpyrifos and its metabolites with pyridine ring were identified by EC/MS and compared with rat liver microsome incubates. However, transformation products of chlorpyrifos without the pyridine ring (diethylthiophosphate, diethylphosphate, or monoethylthioophosphate) were found inactive to glucosylation and glutathione conjugation. In the case of fluopyram, monohydroxyl fluopyram was found to conjugated actively with both glutathione and glucoside. In general, seven bioconjugates of model pesticides’ phase I metabolites with glutathione and glucoside were identified by this non-microsomal approach. The conjugates produced by EC/MS were comparable with rat liver microsome incubates. Compared to in vitro and in vivo, the use of an electrochemical flow-through cell saves time and enable matrix free detection of biotransformation products. EC/MS enables observation of the real-time formation of conjugates by recording the respective mass voltammograms. The work could be an alternative approach to study drug/agrochemical bioconjugates for chemical industries.

## Figures and Tables

**Table 1:** Retention time (t_R_) and MS/MS product ions measured by LC-MS/MS and δm/m measured by TripleTOF of GSH and glucoside (Glc) adducts of CPF and FLP oxidative products using BDD as WE.

Abbreviation/Symbol	t_R_ (min)	Calculated [M + H]^+^	Product Ions by QTRAP-MS/MS	Measured [M + H]^+^	δm/m (ppm)	Type of Conjugate
C1	19.5	487.0007	308, 291, 180, 179, 205, 233	487.0030	4.7	TCP + GSH – H_2_O
C2	20.2	469.0346	469, 451/453, 433, 437, 405	469.0312	1.3	TCP + GSH – HCl
C3C5	21.530.4	621.0407	621, 603/605, 564, 593, 541, 469, 314, 288, 171, 154	621.0534	4.8	CPF + GSH – HCl
C4	23.3	605.0635	605, 564, 587, 573, 569, 555, 540, 339, 327, 298, 251, 154	605.0520	2.2	Oxon +GSH – HCl
C6	29.6	626.9704	609, 538, 369, 322, 308, 231	627.0068	−7.2	U
C7	15.6	359.9803	360, 362, 364, 324, 198, 180	359.9891	−5.2	TCP + Glc – H_2_O
C8	18.4	478.0431	316, 163, 153, 137, 162	478.0410	6.5	Oxon + Glc – HCl
C9	20.5	494.0203	515, 314, 171, 163	494.0299	−3.2	CPF + Glc – HCl
C10 *	23.84	702.1280	705, 513, 429, 395, 308, 173, 145, 129, 291, 179, 162	702.1832	−5.1	Hydroxyl FLP + GSH – H_2_O
C11 *	28.9	701.2423	703, 413, 395, 307, 289, 573, 173, 145, 129	701.2093	−4.3	Hydroxyl FLP + n-Glc – H_2_O

U-unidentified, * conjugates of FLP TPs
